# A Magnetically Coupled Electromagnetic Energy Harvester with Low Operating Frequency for Human Body Kinetic Energy

**DOI:** 10.3390/mi12111300

**Published:** 2021-10-22

**Authors:** Xiang Li, Jinpeng Meng, Chongqiu Yang, Huirong Zhang, Leian Zhang, Rujun Song

**Affiliations:** 1 School of Mechanical Engineering, Shandong University of Technology, Zibo 255049, China; lixiangsdut@163.com (X.L.); mengjinpengvip@126.com (J.M.); yangcq@sdut.edu.cn (C.Y.); fdzhanghr@163.com (H.Z.); ziaver@163.com (L.Z.); 2 Shenzhen Research Institute of City University of Hong Kong, Shenzhen 518057, China

**Keywords:** vibration energy harvesting, electromagnetic energy harvester, magnetic coupling, human body kinetic energy

## Abstract

In this paper, a magnetically coupled electromagnetic energy harvester (MCEEH) is proposed for harvesting human body kinetic energy. The proposed MCEEH mainly consists of a pair of spring-connected magnets, coils, and a free-moving magnet. Specifically, the interaction force between the magnets is repulsive. The main feature of this structure is the use of a magnetic-spring structure to weaken the hardening response caused by the repulsive force. The magnetic coupling method enables the energy harvester system to harvest energy efficiently at low frequency. The MCEEH is experimentally investigated for improving energy harvesting efficiency. Under harmonic excitation with an acceleration of 0.5 g, the MCEEH reaches resonance frequency at 8.8 Hz and the maximum output power of the three coils are 5.2 mW, 2.8 mW, and 2.5 mW, respectively. In the case of hand-shaking excitation, the generator can obtain the maximum voltage of 0.6 V under the excitation acceleration of 0.2 g and the excitation frequency of 3.4 Hz. Additionally, a maximum instantaneous power can be obtained of about 26 mW from the human body’s kinetic energy.

## 1. Introduction

In recent years, the disadvantages of chemical batteries as power supply are obvious. These kinds of shortages including low-level capacity and lifespan, heavy metal pollution, etc. Nowadays, there is a new field that has gained broad concern, and that is the conversion of environmental energy into electric energy [1,2]. These energy conversion technologies can avoid the aforementioned disadvantages of chemical battery power and reduce the dependence of small electronic devices on batteries [3,4,5]. According to the conductive mechanism, the conversion can be divided into piezoelectric [6,7,8,9], electromagnetic [10,11,12,13,14], and electrostatic [15,16,17,18]. The electromagnetic vibration energy harvester has simple structure, small internal resistance, high current, and easy processing compared to other types of energy harvesters, thus receiving more and more research and attention [19,20,21,22]. Conventional electromagnetic generators generally include stators, rotors, bearings, etc. Their complex structure is not conducive to miniaturization [23]. Therefore, researchers have developed a new type of electromagnetic energy harvester. For example, Saha et al. [24] designed an electromagnetic energy harvester (EMEH), which was composed of magnets fixed at the ends of the cylinder, a magnet suspended in the middle of the cylinder by the repulsive force, and a coil wound on the outer surface of the cylinder. The middle magnet can move freely. When the cylinder is subjected to external vibration excitation, the central magnet will vibrate up and down, and the current will generate in the coil. Mann et al. [25,26] designed a novel energy harvesting device that used magnetic restoring forces to harvest energy from the nonlinear oscillations. In addition, Mann innovated and created a nonlinear energy harvester with a bistable potential well. Lee et al. [27] designed an electromagnetic energy harvester that could generate higher output power at a lower frequency. Foisal et al. [28] proposed two kinds of electromagnetic multi-frequency converter array models and used the magnetic spring technique to harvest energy from low-frequency vibrations. Fan et al. [29,30] presented a monostable electromagnetic energy harvester. The energy harvester has a typical softening response and can move the working frequency band to the left. It is very suitable for extracting energy from ultra-low frequency excitation. Further studies by Fan also presented a two-degree-of-freedom (2-DOF) electromagnetic energy harvester (EMEH). Both experiments and simulation verification results showed that the energy harvester can increase the output power and expand the working bandwidth. Zhu et al. [31] investigated a broadband compact electromagnetic energy harvester with a coupled bistable structure. Compared with conventional bistable and linear energy harvesters, this harvester can generate a larger power output. Masana et al. [32] investigated the relative performance of the energy harvester under different monostable and bistable conditions. Results show that the bistable harvester with shallow potential well produced large power in the low-frequency range. Munaz et al. [33] developed an electromagnetic energy harvester using a multi-pole magnet to obtain high power output within a limited volume. Halim et al. [34] designed a frequency conversion harvester that used the collision of small balls to convert low-frequency vibration to a high-frequency vibration. However, energy loss existed in the collision process.

Accordingly, the above harvester has not been studied together with the magnetic levitation structure under magnetic spring structure. Consequently, we propose a magnetically coupled electromagnetic energy harvester (MCEEH) to harvest vibration energy at low-frequency. The proposed MCEEH mainly consists of a pair of spring-connected magnets, coils, and a free-moving magnet. Specifically, the interaction force between the magnets is repulsive. The main feature of this structure is the use of a magnetic-spring structure to weaken the hardening response caused by the repulsive force. This MCEEH has the advantages of adjustable working frequency and high low-frequency energy harvest efficiency. Moreover, this paper also studies the effects of excitation frequency, excitation acceleration, and spring stiffness on the efficiency of energy harvesters under harmonic excitation. The human body kinetic energy experiment was conducted to expand the application of electromagnetic energy harvesters, including: hand-shaking excitation experiment, leg excitation experiment, and backpack experiment. The experimental results show that the MCEEH can be used to power various microelectronic products, e.g., wrist watches, sensors, hearing aids, etc.

## 2. Physical Model and Experimental Method

**Figure 1:**
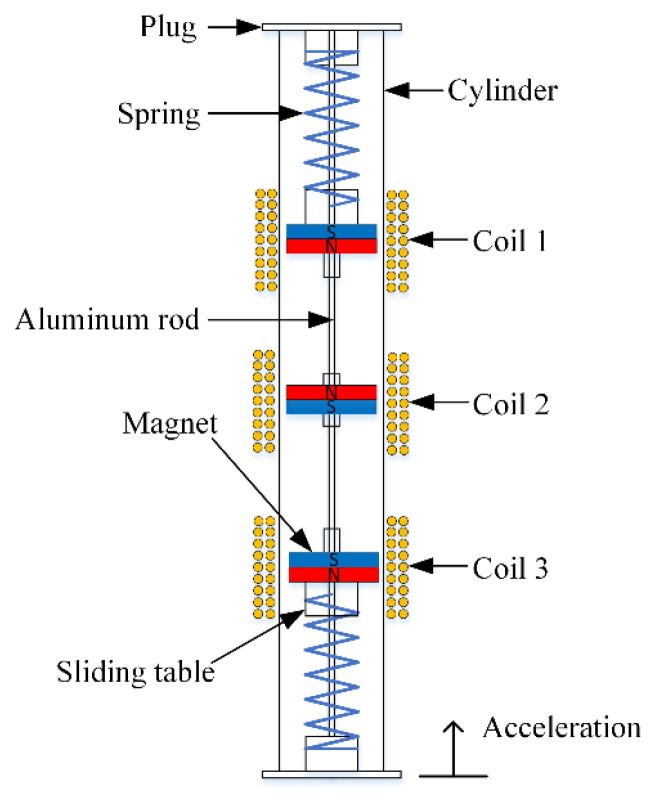
Schematic diagram of the magnetically coupled electromagnetic energy harvester.

In this paper, a magnetically coupled electromagnetic energy harvester is proposed and investigated, as shown in Figure 1. The energy harvester was composed of a cylinder, magnet, spring, sliding table, plug, and aluminum rod. The plug was fixed at the end of the cylinder, and two aluminum rods were fixed at the center of the plug. Furthermore, one end of the spring was fixed to the plug and the other end was fixed to the slide. The sliding table was hollowed and slid freely across the aluminum rod. The aluminum rod connected the whole device internally. Three groups of magnets were placed on the sliding table, two of which were placed on the sliding table at each end and the other central magnet was levitated by the repulsive force of the two magnets from both sides. Three groups of coils were wrapped around the outer surface of the cylindrical cavity.

**Figure 2:**
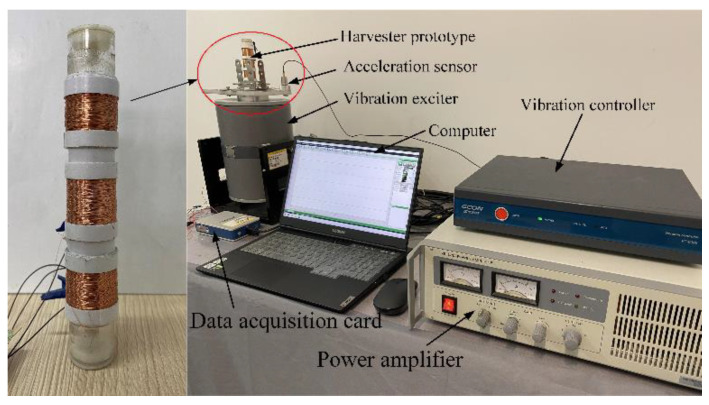
Experimental platform and data acquisition system.

Based on the physical model in Figure 1, the experimental prototype of the MCEEH and test platform was fabricated and assembled respectively, as showed in Figure 2. In detail, the length, outer diameter, and wall thickness of the cylinder were 120 mm, 24 mm, and 2 mm, respectively. Three sets of 800 coils were wrapped around the outside of the cylinder, and the diameter of the coil was 0.12 mm. The internal resistance of the coil and resistance of the external resistance had the same resistance value of 220 Ω. The magnet was round with a hole of about 5 mm. The diameter and thickness of the magnet were 20 mm and 5 mm, respectively. The residual magnetic induction density was 1.32 T. The masses of the upper, middle, and lower magnets were 30 g, 10 g, and 30 g, respectively. The elastic stiffness of the spring was 29.16 g/mm. The experimental system consisted of a vibration exciter, power amplifier, vibration controller, data acquisition card, acceleration sensor, and computer. The acceleration sensor was mounted on the aluminum base plate with a permanent magnet. The data acquisition card was connected to the computer to collect the experimental data such as the real-time voltage at both ends of the load resistor of the MCEEH device. It is worth noting that the cylinder was vertically installed on the bottom plate in the experiment. The output performance of the energy harvester device was investigated by changing the excitation frequency, excitation acceleration, and spring stiffness, respectively.

## 3. Results and Discussion

### 3.1. Electrical Output under Harmonic Excitations

**Figure 3:**
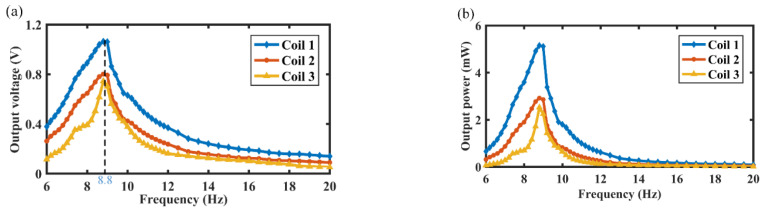
The curve of the MEECH output with excitation frequency: (**a**) the curve of the output voltage with the excitation frequency; (**b**) the curve of the output power with the excitation frequency.

**Figure 4:**
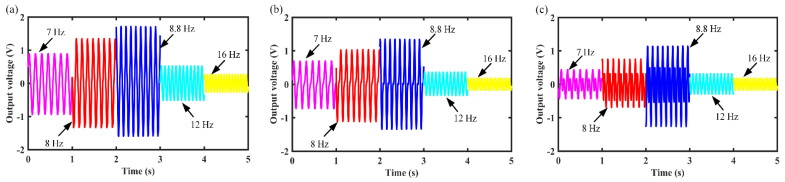
Output voltage of the MCEEH at different frequencies: (**a**) coil 1; (**b**) coil 2; (**c**) coil 3.

This section describes the output characteristics of the MCEEH under vibration excitation. Experimental investigation of the output characteristics of the MCEEH used the test platform in Figure 2. First, the output voltage and output power of the MCEEH were tested with varied excitation frequencies. The excitation acceleration was 0.5 g. Figure 3a is the curve of the output voltage of the MCEEH with the excitation frequency, and Figure 3b is the curve of the output power of the MCEEH with the excitation frequency. It is shown in Figure 3 that the output voltage and power of the MCEEH increased and then decreased with the increase of excitation frequency. When the frequency was 8.8 Hz, the MCEEH reached the resonance frequency. The maximum output voltages of coil 1, coil 2, and coil 3 were 1.05 V, 0.8 V, and 0.75 V, respectively. Obviously, the maximum output power of coil 1, coil 2, and coil 3 was 5.2 mW, 2.8 mW, and 2.5 mW, respectively. The MCEEH can effectively harvest energy from low-frequency vibrations. Figure 4 shows the output voltage of coil 1, coil 2, and coil 3 at excitation frequencies of 7 Hz, 8 Hz, 8.8 Hz, 12 Hz, and 16 Hz, respectively. As shown in Figure 4, the output voltages of coil 1, coil 2, and coil 3 were the highest when the resonance frequency was 8.8 Hz. When any magnet vibrated, the other two groups of magnets showed a significant vibration response resulting in a simultaneous voltage output. Therefore, the magnetic coupling method enabled the energy harvester system to harvest energy efficiently at low frequency.

**Figure 5:**
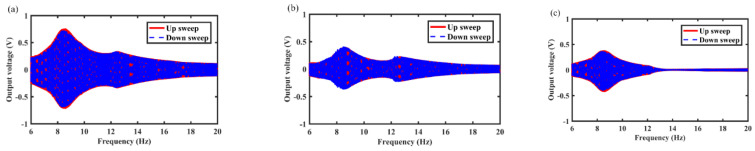
Frequency sweep response of the MCEEH with excitation acceleration of 0.3 g: (**a**) coil 1; (**b**) coil 2; (**c**) coil 3.

**Figure 6:**
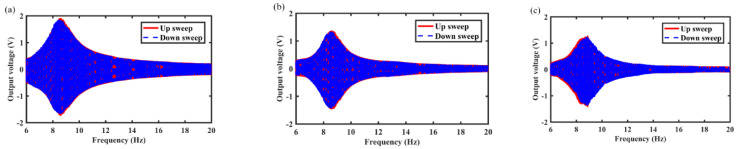
Frequency sweep response of the MCEEH with excitation acceleration of 0.5 g: (**a**) coil 1; (**b**) coil 2; (**c**) coil 3.

**Figure 7:**
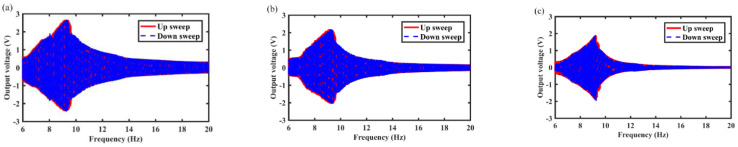
Frequency sweep response of the MCEEH with excitation acceleration of 0.7 g: (**a**) coil 1; (**b**) coil 2; (**c**) coil 3.

The output voltage curves of the MCEEH at different excitation accelerations of 0.3 g, 0.5 g, and 0.7 g are shown in Figure 5, Figure 6 and Figure 7, respectively. It can be found that the resonance frequencies were about 8.6 Hz, 8.8 Hz, and 9.6 Hz at excitation accelerations of 0.3 g, 0.5 g, and 0.7 g, respectively. As can be seen from Figure 5, Figure 6 and Figure 7, the voltage curves for upward and downward frequency sweep operations were almost identical. Hardening response can be observed in upward and downward sweep operations. Increasing excitation acceleration can enhance the output voltage and improve the energy harvest efficiency at low frequency. The reason for this phenomenon was that the displacement of the center magnet increased and the center magnet can be moved closer to the magnets at both ends, increasing with the excitation acceleration. Enhanced coupling between the magnets increased the output voltage. Repulsion between the center magnet and the magnets at both ends caused the hardening response and the resonance frequency right shift. Nevertheless, the magnetic-spring structure weakened the hardening response. Therefore, there was no significant change in resonance frequency at accelerations of 0.3 g and 0.5 g. As the excitation acceleration increased, the role of repulsive coupling between the magnets in the dynamic response became more and more obvious. The hardening response caused by the repulsion of the magnets was highlighted, which resulting in a large resonance frequency at an acceleration of 0.7 g.

**Figure 8:**
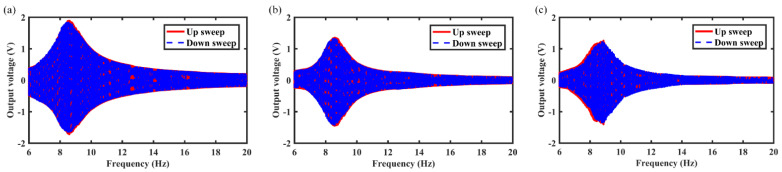
Frequency sweep response of the MCEEH with spring wire diameter of 0.4 mm: (**a**) coil 1; (**b**) coil 2; (**c**) coil 3.

**Figure 9:**
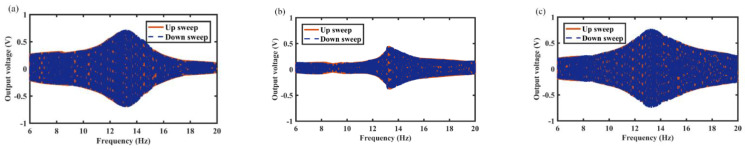
Frequency sweep response of the MCEEH with spring wire diameter of 0.5 mm: (**a**) coil 1; (**b**) coil 2; (**c**) coil 3.

**Figure 10:**
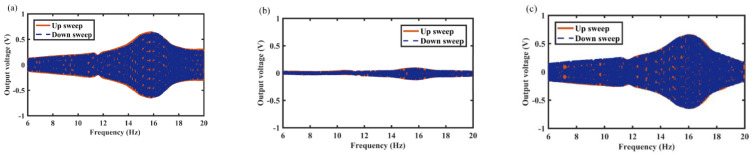
Frequency sweep response of the MCEEH with spring wire diameter of 0.6 mm: (**a**) coil 1; (**b**) coil 2; (**c**) coil 3.

In this section, the effects of spring wire diameter on energy harvest performance are analyzed in detail. For example, Figure 8, Figure 9 and Figure 10 show the variation of output voltage of the MCEEH versus excitation frequency at spring wire diameters of 0.4 mm, 0.5 mm, and 0.6 mm, respectively. All the excitation accelerations were 0.5 g. The spring wire diameter has a positive correlation with spring stiffness resulting in the resonance frequency of the MCEEH increasing. As displayed in Figure 8, the resonance frequency was 8.8 Hz, and the maximum output voltages of coil 1, coil 2, and coil 3 were 1.8 V, 1.35 V, and 1.2 V, respectively. According to Figure 9, the resonance frequency was 13.3 Hz, and the maximum output voltages of coil 1, coil 2, and coil 3 were 0.66 V, 0.4 V, and 0.72 V, respectively. It is depicted in Figure 10 that the resonance frequency was 15.9 Hz and the maximum output voltages of coil 1, coil 2, and coil 3 were 0.62 V, 0.1 V, and 0.65 V, respectively. It can be seen from Figure 8, Figure 9 and Figure 10 that with the increase of the spring wire diameter, the output voltages of coil 1, coil 2, and coil 3 all decreased, especially for coil 2. The reason for this phenomenon was that the free vibration of the center magnet was suppressed and the coupling vibration between the center magnet and the magnets at both ends was correspondingly weakened, as the spring stiffness increased. Thus, the output voltage was reduced.

### 3.2. Electrical Output under Hand-Shaking Excitation

**Figure 11:**
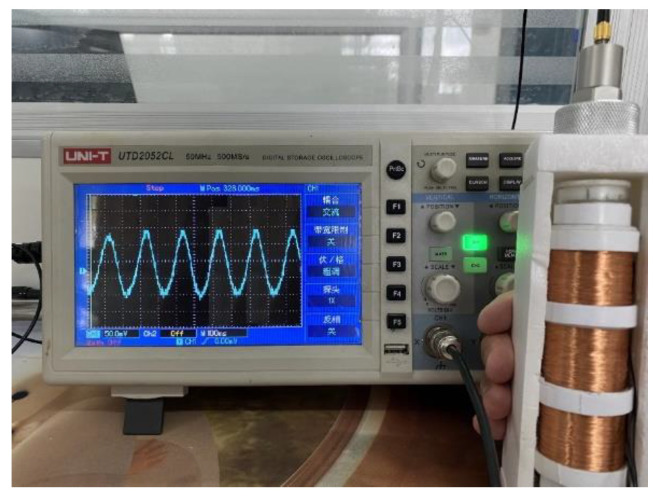
Picture of the hand-shaking test.

**Figure 12:**
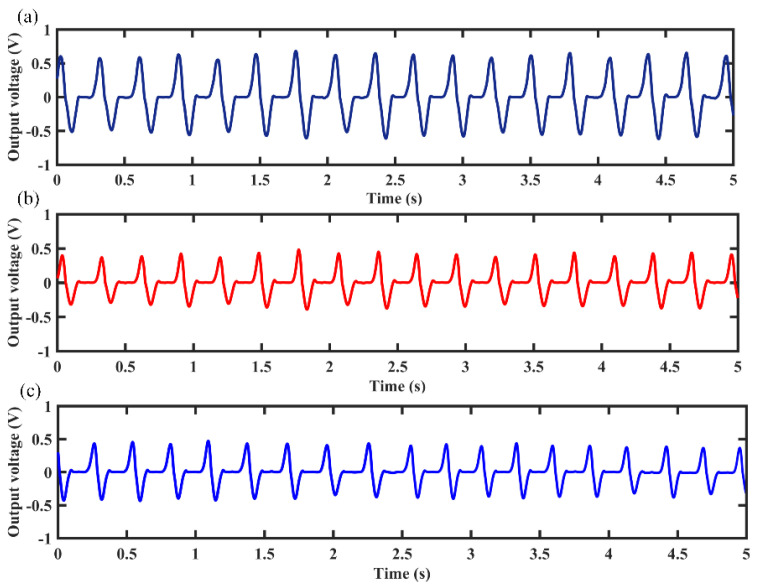
Electrical output under hand-held excitation: (**a**) output voltage of coil 1; (**b**) output voltage of coil 2; (**c**) output voltage of coil 3.

In daily activity, the human body can produce kinetic energy in the process of movement. This human body kinetic energy can be harvested by some devices and converted into electrical energy to power portable microelectronic devices. However, human motion is a low frequency and high amplitude vibration, and a free-moving magnet in the MCEEH can improve the efficiency of obtaining energy from human motion. To verify the feasibility of the MCEEH for powering portable microelectronic devices, the hand-shaking excitation experiment was conducted using the MCEEH. The MCEEH was fixed in the holding device, as shown in Figure 11. During the experiment, the device was held by an adult male. During hand-shaking excitation, the excitation acceleration and excitation frequency were approximately 0.2 g and 3.4 Hz, respectively. The real-time voltage output is shown in Figure 12, the peak voltages generated by coil 1, coil 2, and coil 3 were 0.6 V, 0.4 V, and 0.4 V, respectively.

### 3.3. Electrical Output under Human Motion Excitation

**Figure 13:**
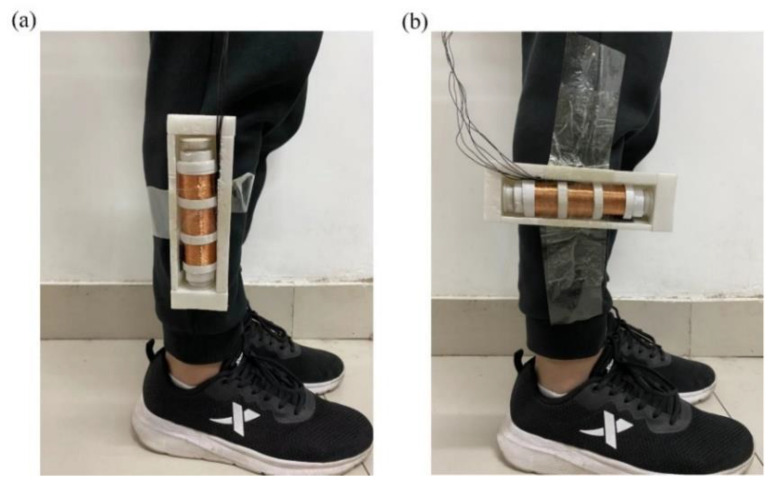
Picture of the human motion excitation test: (**a**) parallel to the leg; (**b**) vertical to the leg.

The MCEEH device was installed on the human body to perform human excitation energy harvest experiments. During the experiment, the energy harvest device was installed on the lower extremity of an adult male with a height of 180 cm. The installation was divided into two: vertical to the leg and parallel to the leg, as shown in Figure 13. In the experiment, the experimenter moved at a constant speed of 3 km/h and 6 km/h, and the voltage generated during the movement was harvested.

**Figure 14:**
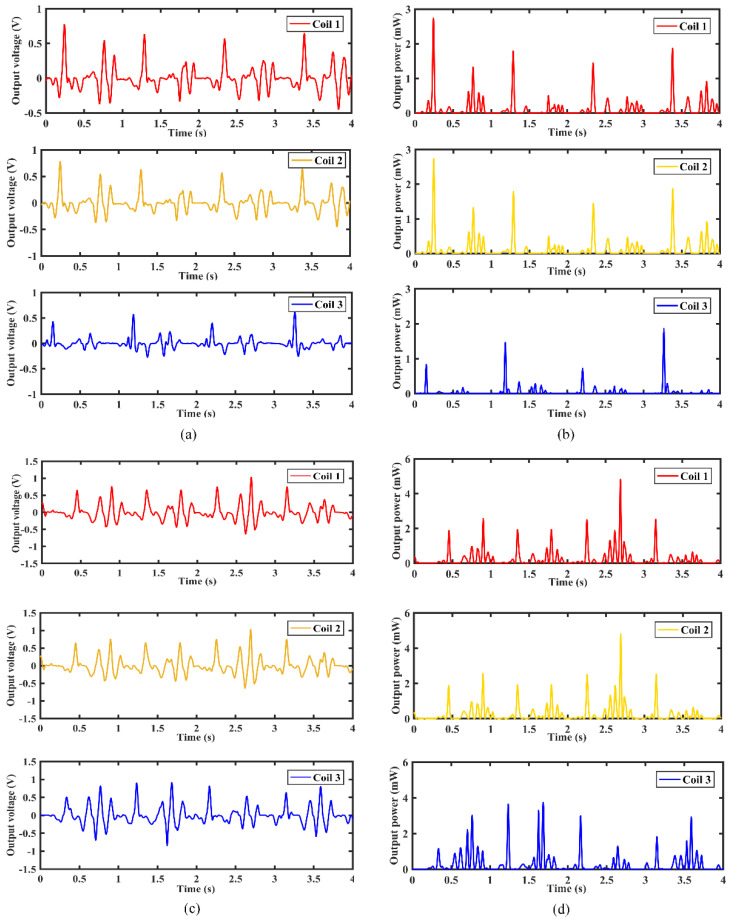
Output of the MCEEH installed parallel to the leg: (**a**) output voltage for moving speed at 3 km/h; (**b**) instantaneous power output for moving speed at 3 km/h; (**c**) output voltage for moving speed at 6 km/h; (**d**) instantaneous power output for moving speed at 6 km/h.

Figure 14 shows the output voltage and instantaneous power output response of the MCEEH installed parallel to the leg, respectively. As shown in Figure 14a,b, when the moving speed was 3 km/h, the maximum voltage and maximum instantaneous power reached 0.8 V and 2.7 mW, respectively. It also can be seen from Figure 14c,d, when the moving speed was 6 km/h, the maximum voltage and maximum instantaneous power reached 1.1 V and 4.8 mW, respectively. The voltage had multiple peaks. One peak voltage was generated when the feet touched the ground, and the other peak voltage was generated when the feet were lifted from the ground. With the increase of moving speed, the change of the voltage peak was not obvious. However, the voltage period was significantly reduced. This demonstrated that the MCEEH can effectively harvest energy in high-speed motion in the state of parallel installation.

**Figure 15:**
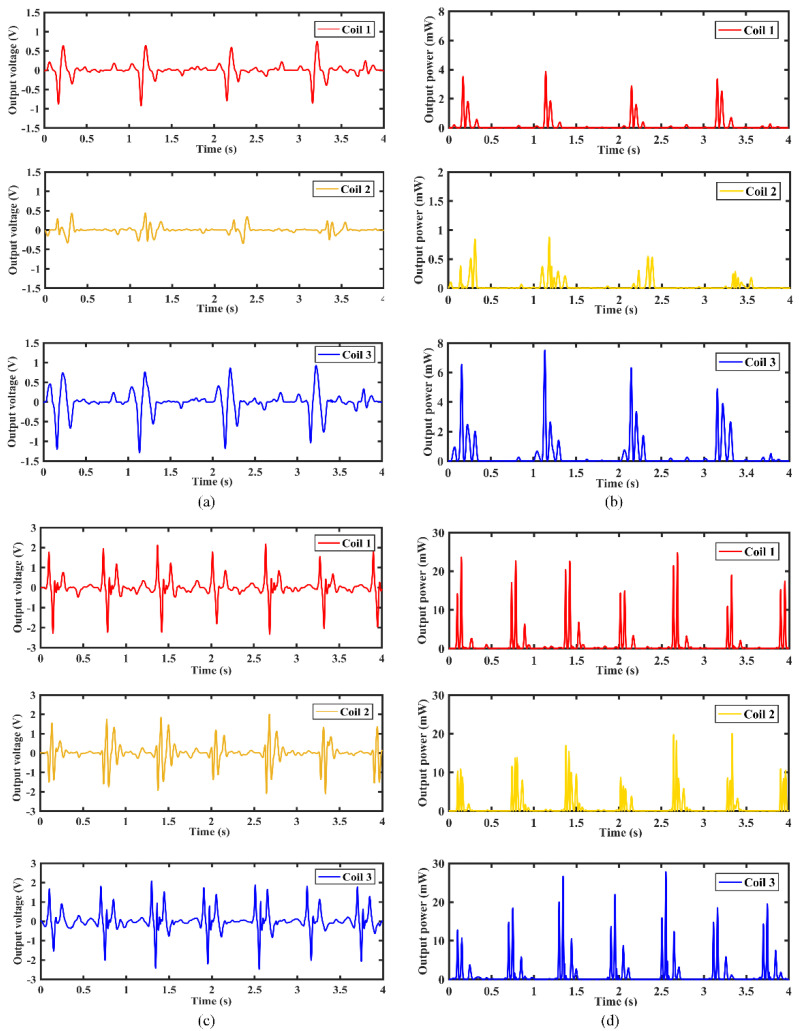
Output of the MCEEH installed vertical to the leg: (**a**) output voltage for moving speed at 3 km/h; (**b**) instantaneous power output for moving speed at 3 km/h; (**c**) output voltage for moving speed at 6 km/h; (**d**) instantaneous power output for moving speed at 6 km/h.

Figure 15 shows the output voltage and instantaneous power output response of the MCEEH installed vertically to the leg. As shown in Figure 15a,b, when the moving speed was 3 km/h, the maximum voltage and maximum instantaneous power reached 0.8 V and 7.4 mW, respectively. It also can be seen from Figure 15c,d, when the moving speed was 6 km/h, the maximum voltage and maximum instantaneous power reached 2.4 V and 26 mW, respectively. The step frequency of 3 km/h was about 0.9 Hz, and the step frequency of 6 km/h was about 1.4 Hz. The voltage increased with higher moving speed. Under the condition of large step frequency, the output voltage was large. This showed that the MCEEH can effectively harvest energy in low frequency in the state of vertical installation.

**Figure 16:**
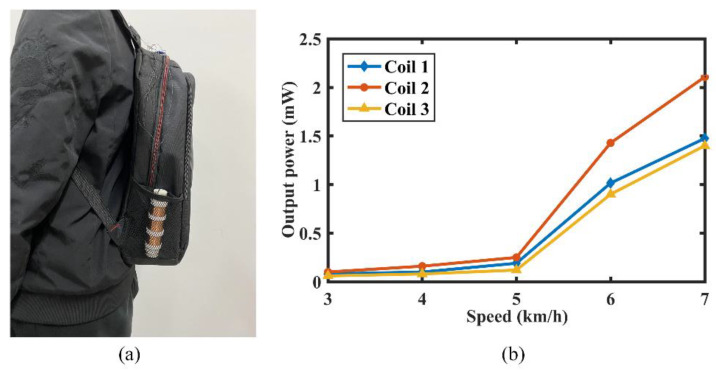
(**a**) Picture of the MCEEH put in a backpack vertically; (**b**) the output power of MCEEH at different speeds.

To verify the performance of the MCEEH without affecting human motion, the MCEEH was placed vertically in a backpack, as shown in Figure 16a. The experimenter traveled with the backpack on a treadmill with constant speeds of 3 km/h, 4 km/h, 5 km/h, 6 km/h, and 7 km/h, respectively. The output power of the MCEEH at different speeds is shown in Figure 16b. The maximum power values of coil 1, coil 2, and coil 3 were 1.5 mW, 2.1 mW, and 1.4 mW, respectively. It is clear from Figure 16 that the output power of the MCEEH increased slightly when the speed varied from 3 to 5 km/h. However, the output power of the MCEEH increased rapidly as the experimenter’s speed changed from 6 to 7 km/h. The reason for this phenomenon was the change in the movement of the experimenter from walking to running.

## 4. Conclusions

In this paper, a magnetically coupled electromagnetic energy harvester (MCEEH) is proposed for converting human body kinetic energy into electric energy. The main novel structure of the MCEEH consists of a pair of spring-connected magnets, coils, and a free-moving magnet. Specifically, the interaction force between the magnets is repulsive. This structure shows a typical hardening response to harmonic excitation. The magnetic-spring structure can weaken the hardening response caused by the repulsive force. A free-moving magnet in the MCEEH can improve the efficiency of obtaining energy from human motion. The effects of excitation frequency, excitation acceleration, and spring stiffness on the vibration response and output voltage of the MCEEH have been experimentally studied. In detail, increasing the excitation acceleration can improve the output voltage. The MCEEH has the advantage of adjustable working frequency and high low-frequency energy harvest efficiency. Increasing the spring stiffness causes the resonance frequency to shift to the right and the output voltage to decrease. The human body kinetic energy experiment was conducted using an MCEEH. For example, the generator can obtain the maximum voltage of 0.6 V during hand-shaking excitation. Furthermore, maximum instantaneous power can be obtained of about 4.8 mW and 26 mW in the case of parallel and vertical orientation to the leg. Maximum power of 2.1 mW can be obtained when placed vertically in the backpack. If the performance of the harvester is further improved, such as the power can be increased by increasing the number of coil turns and using thicker magnets, the MCEEH can be used to power various microelectronic products, e.g., wrist watches, sensors, hearing aids, etc. Accordingly, this paper provides a feasible guideline to improve performance for electromagnetic energy harvesters.
